# Huge vaginal stone: Case report and review of the literature

**DOI:** 10.3389/fsurg.2022.937371

**Published:** 2022-07-01

**Authors:** Jae Yoon Jo, Seon Mi Lee, Jeong Kyu Shin, Won Jun Choi, In Ae Cho

**Affiliations:** ^1^ Department of Obstetrics and Gynecology, Gyeongsang National University Hospital, Jinju, South Korea; ^2^ Institute of Health Sciences, Gyeongsang National University, Jinju, South Korea; ^3^ Department of Obstetrics and Gynecology, Korea University, College of Medicine; ^4^ Department of Obstetrics and Gynecology, Korea University, College of Medicine, Seoul, South Korea

**Keywords:** vaginal stone, spastic quadriplegia, primary vaginal calculus, struvite, recurrent urinary tract infections (rUTIs)

## Abstract

Primary vaginal stones have been rarely reported; the reports that do exist are usually case reports. Because of their low incidence, they are often misdiagnosed. This case report and literature review of a primary vaginal stone presents an assessment of symptoms and common risk factors for vaginal stone formation. A 28-year-old woman with spastic quadriplegia who had been bedridden for most of her life presented to the emergency department for abdominal distension and fever. She had chronic constipation, recurrent urinary tract infections (UTIs), and vaginal discharge. Abdominopelvic computed tomography (CT) was performed and a large stone observed. The vaginal stone was completely removed through the vaginal stump after hysterectomy. Differential diagnoses of vesicovaginal fistula, urethrovaginal fistula, genital anomaly, and ectopic ureter were made by performing several tests using indigo-carmine dye. She recovered from surgery without any complications. There was no recurrence of vaginal stones after 3 months. A biochemical analysis reported that the vaginal stone was 100% struvite. Vaginal stones are caused by repeated infections in an environment in which urine collects gradually. Patients with recurrent UTIs who are bedridden should be able to prevent vaginal stones with periodic gynecological examinations for early diagnosis and management.

## Introduction

Vaginal stones are rare and have been presented only in case reports (1). Stones can occur in the kidney, ureter, bladder, gallbladder, and salivary glands; however, they are commonly found in the urinary system (2). Vaginal stones, also called vaginal calculus, are formed by a mechanism similar to that of urinary stones (3). They are classified as primary or secondary depending on the presence or absence of nidus. Primary vaginal stones result from urine stasis in the vagina; however, secondary vaginal stones form as urine crystalizes around foreign bodies in the vagina (4, 5).

There have been several case reports of vaginal stones; however, they have been found sporadically over the course of approximately 80 years. Therefore, we conducted a review of case reports published in English after the 2000s. While reviewing these case reports, paralyzed patients who spent most of their time bedridden accounted for a high proportion of vaginal stone cases. We aimed to contribute to the knowledge of the origin of vaginal stones among bedridden patients and to prevent this disease by reviewing the literature and sharing our case.

## Case presentation

**Figure 1:**
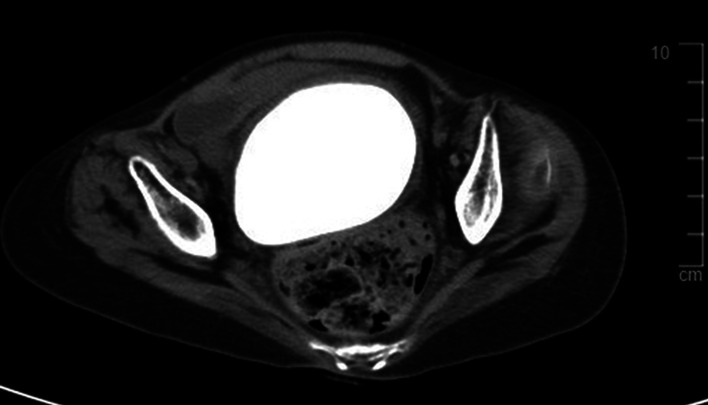
Computed tomography (axial view) show 9.3 cm × 8.0 cm high density mass with smooth margin between bladder and rectum.

**Figure 2:**
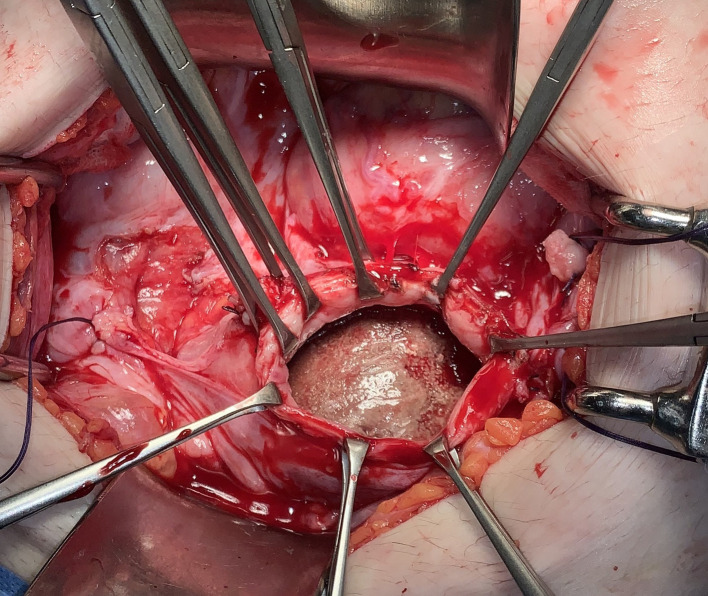
After hysterectomy, stone can see through stump during surgery.

**Figure 3:**
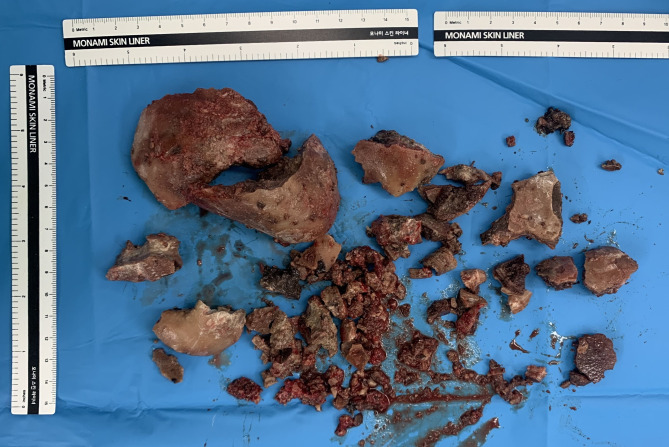
Round laminated vaginal stone was removed through stump after grinding.

A 28-year-old woman presented with fever and abdominal distension to our tertiary hospital emergency department. Abdominopelvic contrast-enhanced computed tomography (CECT) was performed to find the cause of the fever, and a large, hard mass was observed in the vagina (Figure 1). Therefore, she was referred to the gynecology department. The patient was living in a social welfare facility because she had no relatives. Therefore, her caregiver, who was a staff member at the facility, had limited knowledge of the patient's family history and medical history. The caregiver told us that the patient had chronic constipation, recurrent urinary tract infections (UTIs), and vaginal discharge with odor for a prolonged time. The patient had quadriplegia as a result of cerebral palsy when she was young and had undergone laparotomy for peritonitis at approximately 7 years of age. She had severe mental and motor disabilities, joint contractures involving the lower and upper limbs, and spasticity. She spent most of her time bedridden in a supine position and defecating in diapers. Both ovaries and the uterus were normal according to CECT, and there was a 9.3 cm × 8 cm × 6 cm mass compressing the rectum between the bladder and colon; therefore, we suspected that the stone was inside the vagina (Figure 1). During physical examination, her abdomen was distended, her external genitalia was normal, and her hymen was intact. Vaginal discharge was mucoid, yellowish-gray, and had an unpleasant odor. During the speculum examination, a stone-like mass was found within the vagina. Because of her fever, bacteriuria, and pyuria, our diagnosis was UTI accompanied by a vaginal calculus. *Proteus vulgaris* and *Streptococcus anginosus* were found in her urine culture. The UTI was treated antibiotics. We consulted with a urologist and decided to use Holmium laser lithotripsy to cut the vaginal stone into pieces so it could be removed through the vaginal opening. Under general anesthesia, we placed her in the lithotomy position and tried to cut into the vaginal stone with Holmium laser lithotripsy for about 6 h and 45 min; however, the stone was too hard. Consequently, surgery was ended without removing the vaginal stone. She recovered well from surgery and was discharged approximately 1 week later. However, during her follow-up examination at the clinic, it was observed that an edge of the broken stone had stabbed the vaginal wall, resulting in bleeding and inflammation. The secretion worsened and the UTI recurred. After discussion with one of her caregivers, we decided to remove the vaginal stone by performing hysterectomy through laparotomy after treating UTI with antibiotics and daily vaginal irrigation. Under general anesthesia, hysterectomy was performed with a low-midline incision in the lithotomy position. After hysterectomy, the stone was carved out of the vaginal stump using a small drill for approximately 1 h (Figure 2). The vaginal stone was completely removed (Figure 3). To rule out other causes of vaginal stone formation during surgery, the vaginal wall was inspected closely after injection of intravenous indigo-carmine dye. We did not find an ectopic ureter or fistula. The patient was discharged without complications 10 days after surgery. Her caregiver was instructed to maintain the patient's posture upright at least twice per day and to visit the gynecology clinic periodically to prevent the recurrence of vaginal stones. Three months after surgery, the patient had no vaginal stone or UTI recurrence. The vaginal stone weighted 600 g. The biochemical analysis indicated that it comprised 100% struvite.

## Literature review

We searched PubMed and EMBASE for primary vaginal stones and primary vaginal calculus. The search was limited to articles published from January 1, 2000 to March 30, 2022. We included pediatric articles; however, we excluded articles related to secondary vaginal stones. The relevant data summarized in Table 1 were extracted from the case report descriptions. The ages of the patients ranged was from 4 to 69 years. Of a total of 24 patients, 14 presented with urogenital defects or anomalies such as vesicovaginal fistula, urethrovaginal fistula, and vaginal outlet obstruction. Ten out of 24 patients were bedridden with paraplegia or tetraplegia (42%). The periods when patients were bedridden ranged from 5 years to 42 years. The correlation between the length of the bedridden period and the vaginal stone size was not significant. Most case reports included urinary incontinence and recurrent UTIs. Nonspecific symptoms such as fever and stone passage were observed in children and paralyzed individuals. The main causes of vaginal stones are described in Table 1. In 22 of the 24 case reports, urine stagnation was the main cause of vaginal stones. The two excluded case reports, which included vaginal outlet obstruction, assumed that the cause of the vaginal stone was of hematic origin. Among the 24 case reports, six did not describe the stone composition. Forteen of the eighteen case reports that described the composition of the vaginal stones reported that they contained struvite. Other reported components of the stones were carbonate apatite, calcium phosphate, oxalate, and hemosiderine. They were found simultaneously with struvite in three case reports. Four of the 18 case reports that described the composition of vaginal stones did not indicate that the stones contained struvite. In three of the 24 case reports, vaginal stones were removed transperitoneally; in the remaining 21 case reports, episiotomy or hymenectomy was performed to remove the stones vaginally.

**Table 1:** Reports of primary vaginal stones.

Author/year	Age	Immobility/years	Urogenital anatomy	Symptoms	Cause of the stone	Size/component	Treatment
Yoshimura et al., 2000 (11)	11	Yes/11 years	Normal	Vaginal passage of stone, recurrent UTI	Urine retention and infection	4 × 3 cm^2^ /struvite	Transvaginal extraction
Bar-Moshe et al., 2000 (12)	26	No	PVO	Urinary incontinence	Urine retention	5 × 6 cm^2^/not described	Transperitoneal approach
PLAIRE et al., 2000 (13)	4	No	PVO	Urinary incontinence	Urine retention	Not described/ probably struvite	Transvaginal extraction
PLAIRE et al., 2000 (13)	13	No	PVO	Recurrent UTI	Recurrent infection	Not described/ calcium phosphate	Transvaginal extraction
Cetinkurşun et al., 2001 (14)	13	Yes/ 13 years	Normal	Urinary incontinence	Urine retention	4 × 3 cm^2^/struvite (85%) + micronate apatite (15%)	Transvaginal extraction
Ergün et al., 2002 (15)	23	No	PVO	Apareunia	Hematocolpos	1 × 1 cm^2^/hemosiderine + oxalate + Phosphate + carbonate + ammonium	Transvaginal extraction
Malhotra et al., 2004 (3)	21	No	PVO	Apareunia	Hematocolpos	8 × 8 cm^2^/struvite	Transvaginal extraction
Lin et al., 2005 (16)	43	Yes/40 years	Normal	Urinary incontinence	Urine retention	10 × 8 cm^2^/ not described	Transperitoneal approach
Ho and Lin, 2008 (17)	24	No	Transvaginal septum, hypospadias	Dyspareunia	Urine retention	4 × 3 cm^2^/carbonate apatite	Transvaginal extraction
Liu et al., 2008 (18)	14	No	PVO, urethrovaginal fistula	Urinary incontinence, recurrent UTI	Urine retention	2 × 2 cm^2^/struvite	Transperitoneal approach
Oguzkurt et al., 2009 (4)	6	No	Imperforated hymen, urethrovaginal fistula	Abdominal pain, fever	Urine retention	2.5 × 1.5 cm^2^/struvite	Transvaginal extraction
Urbanowicz et al., 2010 (19)	11	Yes/11 years	PVO	Urinary incontinence, recurrent UTI	Urine retention and infection	6.5 × 3 cm^2^/struvite	Transvaginal extraction
Jaspers et al., 2010 (20)	5	Yes/5 years	Normal	Recurrent UTI	Urine retention and infection	3 × 2 cm^2^/struvite	Transvaginal extraction
Chen et al., 2011 (21)	12	No	PVO, vesicovaginal fistula	Dysuria	Urine retention and infection	8 × 7 cm^2^/struvite	Transvaginal extraction
Avsar et al., 2013 (22)	22	Yes/22 years	Normal	Chronic pelvic pain	Urine retention and infection	9 × 7 cm^2^/struvite	Transvaginal extraction
Ikeda et al., 2013 (23)	42	Yes/42 years	Normal	No symptoms	Urine retention	2 × 1.5 cm^2^/struvite (98%) + calcium phosphate (2%)	Transvaginal extraction
Gunes and Uygun, 2014 (24)	11	No	Wide vaginal orifice	Vaginal passage of stone	Urine retention	1.5 × 1.5 cm^2^/struvite	Transvaginal extraction
Castellan et al., 2017 (1)	34	Yes/34 years	Normal	Fever	Urine retention and infection	5.1 × 3.7 cm^2^/struvite	Transvaginal extraction
Tokgöz et al., 2018 (25)	14	Yes/9 years	Normal	Poor appetite	Urine retention and infection	3.8 × 2 cm^2^/struvite	Transvaginal extraction
Wei et al., 2019 (26)	28	No	Urethrovaginal fistula	Frequency, dyspareunia	Urine retention and infection	6 × 5 cm^2^/not described	Transvaginal extraction
Xu and Zou, 2020 (27)	23	No	Urogenital sinus anomaly	Recurrent abdominal pain amenorrhea	Urine retention	8 × 7 cm^2^/not described	Transvaginal extraction
Fedrigon et al., 2020 (28)	61	Yes/15 years	Normal	Vaginal bleeding	Urine retention	10.6 × 8.8 cm^2^/struvite (60%) + calcium phosphate (40%)	Transvaginal extraction
Ranawaka et al., 2020 (29)	12	No	Urogenital sinus anomaly	Recurrent abdominal pain	Urogenital sinus anomaly	1.4 × 0.8 cm^2^/not described	Transvaginal extraction
Kurmanov et al., 2022 (30)	69	No	Vesicovaginal fistula	Vaginal discharge	Urine retention	5 × 0.5 cm^2^/not described	Transvaginal extraction

*PVO, Partial vaginal outlet obstruction.*

## Discussion

The occurrence of vaginal stones is rare and has been reported only in case reports since 1926 (6). Most of the vaginal stones reported so far comprised struvite. Struvite is composed of magnesium ammonium phosphate. It is formed by a combination of two factors: decreased urine volume and infection with bacterial species such as *Proteus*, *Staphylococcus*, *Pseudomonas*, and *Klebsiella*, which increase the urine pH by producing urease (7, 8). Many of the case reports classified vaginal stones as primary or secondary according to the presence or absence of a nidus (6). Secondary vaginal stones are caused by repeated infections caused by foreign bodies such as an intrauterine device (5), surgical gauze (9), or mesh (10) that act as the nidus and promote mineral accumulation. In contrast, primary vaginal stones do not have a nidus. Related case reports are listed in Table 1.

We reviewed the case reports of primary vaginal stones published since 2000. The majority of the case reports described urine stasis in the vagina and recurrent UTIs, which might be important risk factors for the formation of vaginal stones. Vesicovaginal fistula, urethrovaginal fistula, urogenital sinus anomaly, and genital anomaly cause urinary retention in the vagina. Of 25 cases reports (including our case report), 14 cases involved a urogenital anatomic abnormality such as a vaginal outlet obstruction, fistula, or urogenital sinus anomaly. Notably, 11 of the 24 patients reported (including our patient) were bedridden for a long period of time because of disability. In other words, patients with a vaginal stone seem to be bedridden for long periods or have a genitourinary malformation. In particular, the lying position can result in urine retention that is severe enough to form a fistula. During surgery of our patient, we found that the bladder capacity was approximately 50 ml, which is approximately 10-times smaller than that of the adult bladder. Presumably, dissonance between contraction of the bladder detrusor muscle and the urethral sphincter causes decreased bladder capacity and leads to urine leakage. Leaked urine passes through the vaginal wall and stagnates in the vaginal cavity, thus leading to bacterial colonization in the stagnant urine. Because *Proteus* species proliferate in urine, and because the urine pH was 9 in our patient, the infected urine passed to the vagina, resulting in urease production and an alkaline vaginal environment. Ureolysis caused by urease increases ammonia and bicarbonate, thus creating a supersaturated state and resulting in struvite crystal formation (8). Because the enlarged vaginal stone pressed the bladder, the bladder became atrophied, and urine leakage became more frequent. Because the stone remained in the vagina, more frequent UTIs occurred. The patient had no history of child birth, and the stump was expected to be too small after hysterectomy to remove the vaginal stone through laparotomy; therefore, we attempted to remove it through vaginal extraction. However, Holmium laser lithotripsy was unsuccessful because the stone was difficult to break. The second surgery confirmed that our prediction was wrong. The stump and vaginal wall, which were exposed after hysterectomy, were sufficiently dilatated; therefore, the vaginal stone was cut into pieces (Figures 2, 3).

This case report and literature review had several limitations. The vaginal stone in this case did not undergo culture testing; hence, it was inferred through urine culture testing that it was not the primary cause of bacteria. Because only case reports were reviewed and there was no control group for comparison, we could not estimate the general incidence of vaginal stones among nonimmobilized patients or accurately define the risk factors for vaginal stones.

Vaginal stones are rare and have been described only by reports. Therefore, we reviewed other case reports to contribute to the knowledge of vaginal stones in immobilized patients. These patients present to the hospital with nonspecific symptoms because they are not able to express their discomfort or pain. If patients with immobility have recurrent UTIs, then clinicians should perform simple radiography or consult a gynecologist to confirm or dismiss the diagnosis of vaginal stones and prevent their complications.

## Conclusion

When treating bedridden patients, especially those with recurrent UTIs, it is necessary to consider the possibility of vaginal stones because their symptoms are not specific. For women, a gynecologic examination and simple radiography should be considered.

## Data Availability

The raw data supporting the conclusions of this article will be made available by the authors, without undue reservation.
